# The role of medical students in junior doctors’ strikes: A cross-sectional online survey

**DOI:** 10.12688/mep.20227.1

**Published:** 2024-08-01

**Authors:** Shonnelly Novintan, Hannah Okechukwu

**Affiliations:** 1 Southend University Hospital, Westcliff-on-Sea, Essex, SS0 0RY, UK; 2 Imperial College London, London, England, UK

**Keywords:** Strike, education, student, pay restoration, industrial action

## Abstract

**Objectives:**

The British Medical Association announced a successful vote towards industrial action to achieve ‘pay restoration’ on 20 February 2023; with 11 walkout periods occurring in the following months. During industrial action, concerns arose about the role medical students would play and the pressure placed upon them to ‘act up’. The objective of this study was to assess the guidance issued by medical schools and local placement sites during industrial action.

**Design, setting and participants:**

This cross-sectional study collected online survey data between 7 March 2023 and 7 April 2023 from medical students across England.

**Main outcome measures:**

Reports about guidance issued by medical schools and hospital placements.

**Results:**

62% of the medical schools issued guidance stating they were not cancelling clinical placements; of these, 10% said attendance was a personal choice. 17% of medical schools cancelled all clinical placements and 7% did not issue guidance. 52% of medical schools monitored attendance on strike days. 1 medical school and 3 clinical placement sites advertised paid work for students during the industrial action.

**Conclusion:**

The impact industrial action has on medical students has not been examined. Our results show mixed guidance from medical schools that can contradict local placement guidance. This lack of guidance is mirrored in the existing, yet limited, literature. If students feel pressured to perform tasks outside their remit, with inadequate supervision, it can impact patient safety and their license to practice. For the safeguarding of patients, and students, further work is needed to produce standardised guidance during industrial action.

## Introduction

### Background

On 20 February 2023, the British Medical Association announced that 98% of junior doctors in England voted in favour of taking industrial action ^1^. A 72-hour strike was the first act of industrial action and took place between 13 and 16 March 2023. Subsequent industrial action took place between 11 and 15 April 2023, 14 and 17 June, and 13 and 18 July 2023 ^2^. At the time of manuscript publication, there have been a total of 11 rounds of strike action, including a 144-hour walkout beginning on the 3rd January marking the longest duration of strike action taken by junior doctors in the National Health Service (NHS) ^3, 4^. During periods of industrial action, and the subsequent pressure on service provision, concerns arose about the role medical students play and the pressure placed upon them to ‘act up’ ^4^. The impact industrial action has on medical students has not been examined, which demonstrates an interesting avenue of research given that the current industrial action represents the largest junior doctor strike in NHS history.

### Objective

The objective of this study was to assess the guidance issued by medical schools and local placement sites during industrial action.

## Methods

This cross-sectional study collected online survey data from medical students across England between 7 March 2023 and 7 April 2023. The survey contained 7 multiple-choice items with 1 short-answer question. Participants were asked to remove identifying information.

The survey covered participant demographics and medical school guidance issued during strike action. It also provided an opportunity to elaborate on any answers using free-answer text boxes and the option to upload supporting evidence. The use of objective questions also contributed to limiting bias within the dataset. 

Prior to national distribution, feedback on survey design and content was solicited from the research group, composed of current doctors, and incorporated into the final version. This internal pilot testing was used to determine face validity of the questions, alongside assessing for clarity, accuracy, and comprehensiveness. Responses to the survey were collected using Google Forms and were accessed by participants via an electronic link. Informed consent was indicated through completion of the survey following an informed consent statement.

The survey was distributed nationally through Doctor’s Association UK social media platforms and newsletters for data collection, employing a single-stage convenience sampling approach with a goal of having at least one participant representing each institution. Participation was voluntary and respondent anonymity was guaranteed by design.

Inclusion criteria included current medical school students in England on a clinical placement.

After the closing date of the survey, submissions were downloaded as a CSV file and data analysis was completed using chi-squared testing.

### Ethical approval and consent

This project did not require ethical approval as it involved the collection of nonsensitive data, participants were completely anonymised and from a population not classified as vulnerable, and the survey did not contain material that could cause distress to participants. This decision also aligns with Imperial College London's ethical guidelines. This project fell under the service evaluation criteria to define and judge how medical students were treated during industrial action. This was checked per the NHS Health Research Authority REC review tool. To maintain the anonymity of participants, informed consent was obtained through the completion of the survey following an informed consent statement at the start of the form.

## Results

### Guidance issued

57 medical students responded representing 26 of the 28 medical schools in England that are fully registered and accepted by the General Medical Council (GMC). 3 of the 9 medical schools that are under review by the GMC were also represented.

Of the participants, 39% (n=22) were in their fourth year, 32% (n=18) were in their fifth year and 23% (n=13) were in their third year.

During the industrial action, 62% (n=18) of the medical schools issued guidance stating that they were not cancelling clinical placements. Of these, 10% (n=3) said it was a personal choice if they chose to not attend. One medical school stated that the onus was on the placement leads to enforce placement attendance.

17% (n=5) of medical schools cancelled all clinical placements and a further 10% (n=3) of medical schools cancelled hospital clinical placements only.

7% (n=2) of medical schools did not issue guidance to their medical students.

52% of medical schools (n=15) monitored the attendance of students on strike days and three medical schools stated that inadequate time on placement might lead to implications in the progression of training.

### Paid work

One medical school advertised paid work for medical students during the industrial action: Plymouth University Peninsula Schools of Medicine and Dentistry. Three clinical site placements belonging to the following medical schools advertised paid work for students during strike action: Plymouth University Peninsula Schools of Medicine and Dentistry, Queen Mary University of London and University of Leeds School of Medicine.

## Discussion

Industrial action taken by junior doctors has a deliberate impact on patient care, staffing effects and financial costs, and can have professional consequences for doctors themselves; however, the impact of industrial action on medical students is often overlooked.

Medical students in their clinical years often work long hours on placement with little to no compensation and reduced training opportunities due to current service pressures. During strikes, medical students may be asked and feel pressured to perform tasks beyond their capability or without proper supervision. Although they may be nearing graduation, if they feel pressured to perform tasks outside their remit, be it in a paid role or not, it can have consequences for patient safety and their license to practice.

The role of medical students during industrial action is not well documented. The British Medical Association has provided official advice ^5^. One article commenting on the strikes briefly highlights the disparity between medical school-issued guidance, referencing the experiences of students and informal advice of a consultant from Twitter ^4^.

Internationally, medical students have been shown to support doctor strikes, particularly if pay and poor patient care were the reasons behind the strike, but their role during industrial action was not specified ^6, 7^.

Similarities were demonstrated during the Irish Medical Organisation industrial action in 2013 where the majority of medical students were not given guidance, which led to some students joining the picket line. Students were placed in a difficult position of having to choose between supporting their future colleagues and prioritising their education. An inter-medical school collaborative policy was recommended as a result, but this did not come to fruition ^7^.

This poses the question of who is responsible for safeguarding medical students should they be pressured to ‘act up’ during industrial action. In principle, medical schools should be responsible for their students, but this becomes challenging when they do not publish guidance ahead of industrial action or when their guidance differs from individual placement guidance. If individual trusts and/or hospitals offer paid roles for medical students during strikes, it could imply that the trust would hold responsibility. Notably, final year medical students would also hold GMC provisional registration and be held accountable to both their Trust and the GMC. Ultimately, medical students are themselves responsible for knowing their capabilities and competencies, but it is worth taking into consideration that industrial action to this extent is unprecedented and might have placed heavier pressure on students to ‘act up’, especially in these paid roles and in the absence of medical school guidance.

One limitation of this survey was the number of medical schools that were represented, as not every medical school in England had a student who participated. Secondly, the use of a survey means the data collected is susceptible to participation bias, however, to mitigate this fact the survey was shared over multiple channels. The results from this study are also only applicable to medical schools in England as the strikes only apply to England. Finally, a larger sample size would have allowed for greater generalisability of results.

As highlighted by our results, there is mixed guidance from medical schools, which in some instances has been contradictory to local placement guidance. This lack of guidance is mirrored in the existing, yet limited, literature. For the safeguarding of medical students and patients, further work is needed to produce standardised guidance during industrial action.

## Data Availability

The underlying and extended data (survey questions) were obtained from Doctors Association UK and can be accessed upon request via their email address: contact@dauk.org.
