# Supplementary material for: Navigating Industrial Action: The Guidance for Medical Students During Resident Doctor Strikes in England
Source: MedEdPublish (2016). 2024 Dec 24;14:60. Originally published 2024 Aug 1. [Version 2] doi: 10.12688/mep.20227.2 (PMC11809142; doi:10.12688/mep.20227.2)
Supplement: Peer review history

# Reviewer report: Reviewer response for version 2

**DOI:** 10.21956/mep.22328.r41061

**Published:** 2025-02-07

**Authors:** Tyler Bland

**Affiliations:** 1 University of Idaho, Moscow, USA

**Summary of study:**

 This study examined the guidance provided by medical schools and clinical placement sites in England during the 2023 industrial action by resident doctors. The authors performed a cross-sectional survey conducted between March and April 2023 and gathered responses from medical students across fully registered medical schools in the UK. Findings revealed inconsistent guidance, with 62% of schools maintaining placements but varying in attendance policies, while 17% canceled all placements. Additionally, 52% of schools monitored attendance, and some placement sites advertised paid roles for students. The authors conclude with concerns over students feeling pressured to perform tasks beyond their capabilities, which could compromise patient safety and their professional standing.

**Reviewer comments:**

**1. Are sufficient details of methods and analysis provided to allow replication by others?**

- The authors do not specify how they analyzed the open-ended question from the survey. This should be clarified.
- While the authors mention performing chi-squared analysis, this analysis is not presented in the manuscript. It is also unclear which data were used for this analysis.
- The authors should specify how many respondents participated from each medical school. Were there multiple respondents per school, or just one? Additionally, for schools with multiple respondents, it is important to analyze whether their responses were consistent or if there were discrepancies. This should be explicitly stated and explained.

**2. If applicable, is the statistical analysis and its interpretation appropriate?**

- The authors mention performing a chi-squared analysis, but they do not clarify which data were analyzed, nor do they present the results in the manuscript. This should be addressed.
- The results section would benefit from presenting the data in a table.

**3. Have any limitations of the research been acknowledged?**

- A significant limitation of this study is the small sample size (n=57), which should be clearly acknowledged.
- Another limitation is the potential small sample size from each university. The manuscript states that 57 responses were collected across 26 medical schools, but the number of respondents per school is not detailed. It is possible that most schools had only one respondent (n=1), while a single school may have had a much larger number of respondents (e.g., n=32). Schools with more respondents are more likely to provide data that reflect the ground truth, which should be considered. This data should also be added to the results section.
- Another limitation is the subjective nature of the data. Participants may have misremembered or overlooked communications from their universities. As a medical school professor, I have observed students frequently forgetting that they receive daily communications. While the authors included an option for participants to upload corroborating evidence to their survey response, the results of these uploads are not presented or discussed. Additionally, the authors should consider why they did not directly reach out to universities to obtain official communications sent to students.

**4. Title Recommendation:**

- Apologies for another title change suggestion, but this study primarily examines students' perceptions of the guidance they received. Unless the authors provide corroborating evidence from the respondents or universities such as official school communications, the manuscript only reflects students' recollections. In legal terms, this constitutes hearsay, as it relies on students recounting what they believe their schools told them. The title should be revised accordingly to reflect this distinction.

These points should be addressed before acceptance for indexing.

Have any limitations of the research been acknowledged?

Partly

Is the work clearly and accurately presented and does it cite the current literature?

Yes

If applicable, is the statistical analysis and its interpretation appropriate?

Partly

Are all the source data underlying the results available to ensure full reproducibility?

Yes

Is the study design appropriate and is the work technically sound?

Yes

Are the conclusions drawn adequately supported by the results?

Yes

Are sufficient details of methods and analysis provided to allow replication by others?

Partly

Reviewer Expertise:

Medical education, Curriculum design and evaluation

I confirm that I have read this submission and believe that I have an appropriate level of expertise to confirm that it is of an acceptable scientific standard, however I have significant reservations, as outlined above.

# Reviewer report: Reviewer response for version 2

**DOI:** 10.21956/mep.22328.r40995

**Published:** 2025-02-07

**Authors:** Joanna Jane Garstang

**Affiliations:** 1 University of Birmingham College of Medical and Dental Sciences, Birmingham, UK

The article has already been significantly revised and now reads well.  I have no comments on the main article.

 The discussion should also refer to the limitation that the survey is a snapshot in time at the start of the strikes.  Medical schools may have issued or changed guidance later in the strike - this has not been captured.  I doubt anyone expected the strike to last as long as it did.

 I would suggest that the numbers as well as % are given in the abstract as it is a small sample.

Have any limitations of the research been acknowledged?

Yes

Is the work clearly and accurately presented and does it cite the current literature?

Yes

If applicable, is the statistical analysis and its interpretation appropriate?

Yes

Are all the source data underlying the results available to ensure full reproducibility?

Yes

Is the study design appropriate and is the work technically sound?

Yes

Are the conclusions drawn adequately supported by the results?

Yes

Are sufficient details of methods and analysis provided to allow replication by others?

Yes

Reviewer Expertise:

Community child health

I confirm that I have read this submission and believe that I have an appropriate level of expertise to confirm that it is of an acceptable scientific standard.

# Reviewer report: Reviewer response for version 2

**DOI:** 10.21956/mep.22328.r40678

**Published:** 2025-01-02

**Authors:** Surinder Singh

**Affiliations:** 1 University College London, London, England, UK

Thank you for asking me to look at this paper again.  I am pleased that the title of the paper has been appropriately amended. In fact, I think the title is very clever and the introduction of the word *navigating* hits 'the nail on the head'.

 I acknowledge that indexing of the survey is constrained by the ownership by DAUK - but access is possible presumably by e-mailing them.

 With regards to the discussion and who holds responsibility (see 6th paragraph). The authors state that

*"Ultimately, medical students are themselves responsible for knowing their capabilities and competencies, but it is worth taking into consideration that industrial action to this extent is unprecedented...."*

 I do agree with this but to solely rely on a student's self-assessment of capability and competence may be insufficient. I also think it requires quite a lot of insight on the side of the student.  Importantly, I do not expect the authors to change anything here - it is just my contribution to the various arguments which are genuinely taxing!

 As far as I can tell the remainder of my queries have been addressed and I think this paper is now ready for Indexing.

Have any limitations of the research been acknowledged?

Yes

Is the work clearly and accurately presented and does it cite the current literature?

Yes

If applicable, is the statistical analysis and its interpretation appropriate?

Not applicable

Are all the source data underlying the results available to ensure full reproducibility?

Partly

Is the study design appropriate and is the work technically sound?

Yes

Are the conclusions drawn adequately supported by the results?

Partly

Are sufficient details of methods and analysis provided to allow replication by others?

Partly

Reviewer Expertise:

Undergraduate Medical Education; Medical Education pertaining to primary care; qualitative and ethnographic studies; qualitative studies around infection diseases

I confirm that I have read this submission and believe that I have an appropriate level of expertise to confirm that it is of an acceptable scientific standard.

# Reviewer report: Reviewer response for version 1

**DOI:** 10.21956/mep.21655.r38267

**Published:** 2024-08-22

**Authors:** Surinder Singh

**Affiliations:** 1 University College London, London, England, UK

Many thanks for asking me to review this paper which I found interesting and covering a relatively unexplored area.

 I will try and provide comments under the various sections of the paper.

**Abstract:**

 Essentially this needs to be re-written in view of my comments below.

 I note that the objectives of this cross-sectional study (see page 1) *was to assess the guidance issued by medical schools and local placement sites during industrial action.*

 I also note the title of the paper - **the role of medical students in junior doctors' strikes: a cross-sectional online survey.**

 My feeling is that there is a slight difference in the objectives of the study and the title of the paper. While there is much overlap between the study objectives and 'the role of students during the strike' - the two aspects are not totally convergent. This non-convergence is heightened by the fact that the results merely report the guidance from medical schools (see also my comments below).

**Background (page 3)**

 This section is fine and points the way to why this research is both topical and also important since the doctor's strikes were the largest ever in NHS history.

**Methods (page 3)**

 This section is fine through I wondered if the questionnaire could have been shown to a selection of medical students, rather than doctors - since it is the students who were the target group?  In the interests of transparency, could the survey be included in an appendix?

 As stated, the survey covered the issue of *medical school guidance issued during strike action *(2 ^nd^ paragraph). I'm presuming that this information was as provided by the medical school at the time of the strike- i.e. it was not for students to interpret how the guidance - where it existed - was to be implemented?

**Ethical Approval:**

 This section is fine except I did wonder about students who were particularly conflicted? For example who would they go to for help/support? I can imagine a scenario of a senior student who could genuinely help during the strike but who is 'in two minds' because he/she is on the side of the doctors  - the professional group they're about to join - yet does not want to harm patients? (please also see my comments within the discussion - below).

**Results:**

 This is a good section but is limited to the number of respondents and their medical school origins along with the guidance issued from the latter. Noting that N=57, who were the students who did not fit into years 3-5 (numbering 53)?

 Also, it is stated that 18 schools issued guidance that they were not cancelling placements; this should be 64% (not 62% as stated), unless the denominator is wrong?

**Paid Work (page 3):** I wasn't sure why certain medical schools were named here?   In contrast - the majority of the results anonymised the medical schools - for example 5 schools cancelled all clinical placements and a further 3 cancelled hospital clinical placements (see results).

**Discussion:**

**The first paragraph is fine **- and sets the context very well.    

 Perhaps the most important questions here are firstly whether students are asked to do extra tasks while the strike is on and secondly, whether these tasks put students in the invidious position of undertaking them without supervision or working beyond their capacity?

**Interestingly the second paragraph of the discussion highlights the question/issue I mentioned earlier** - that clinical students might feel conflicted about helping out during the doctor's strikes.  I note that paragraph 5 of the discussion does the same with examples from Ireland.

**Paragraph 6 highlights student safeguarding** - in scenarios where pressure is applied for students to 'act up'.  Additionally, it points to possible conflicts between the advice/guidance given by the medical school and that of the *individual placement *(2 ^nd^ sentence) *.*  Was this a finding for it to be mentioned in the discussion or was this a theoretical possibility?  

**The issue of who is ultimately responsible for a paid final-year student if something goes awry **- noting that the student is GMC-registered (provisional), 'employed by the trust' and still under the aegis of a medical school is comprehensively discussed in this paragraph.

**Limitations of study (penultimate paragraph) **- I agree but it is clearly premature to mention generalisability; surely it might be better to state that these are preliminary results and deserve further exploration (which they do in my opinion).

**In summary:**

 Despite my comments I quite liked this paper, however it is not ready for publication just yet. The biggest hurdles in my opinion are threefold:

1. Firstly, try and align the objectives of the study with the title and the overall flow of the paper.
2. Please re-think the results and how they are presented in this section. Additionally address the conflicts issue I highlight above - were these identified?
3. The discussion needs to reflect and align with the objectives of the study and the results with a bit more coherence.

Have any limitations of the research been acknowledged?

Yes

Is the work clearly and accurately presented and does it cite the current literature?

Yes

If applicable, is the statistical analysis and its interpretation appropriate?

Not applicable

Are all the source data underlying the results available to ensure full reproducibility?

Partly

Is the study design appropriate and is the work technically sound?

Yes

Are the conclusions drawn adequately supported by the results?

Partly

Are sufficient details of methods and analysis provided to allow replication by others?

Partly

Reviewer Expertise:

Undergraduate Medical Education; Medical Education pertaining to primary care; qualitative and ethnographic studies; qualitative studies around infection diseases

I confirm that I have read this submission and believe that I have an appropriate level of expertise to state that I do not consider it to be of an acceptable scientific standard, for reasons outlined above.

# Response

**Published:** 2024-10-24

**Authors:** Hannah Okechukwu

**Affiliations:** School of Medicine, Imperial College London, London, England, UK

**Reviewer comments:**

***Title vs objectives: *** We understand the slight ambiguity in the title of the paper compared to the objectives of the study, and have amended out title to more closely reflect the exact goals of our research   

**Response:**

***Methods:*** As the survey is proprietary content of DAUK, we were not able to include it in order to avoid copyright infringement. However, the survey and survey results are both accessible upon request via their email address: contact@dauk.org. The survey covered the issue of medical student guidance during strike action from the perspective of the students themselves as they were our target group for relaying the information on the guidance medical schools were providing them as stated in this section.  

**Reviewer comments:**

***Ethical approval: *** The goal of this study was specifically to assess the guidance provided by medical schools rather than access to further support resources which would be beyond the scope of our research. In terms of the help students can deliver, they are only sanctioned to work within their own capabilities and the onus is on universities to provide clear guidance for students to follow so that they know when they may need to excuse themselves from a given situation. Beyond this, medical students are not officially part of strike action so would be expected to participate in clinical activities as per usual unless directed otherwise by university guidance.  

**Response:**

***Results:*** Of the respondents not mentioned, 3 were in year 2, and 1 was in their intercalation year.  62% is the correct statistic as we stated as we have students of 26 fully registered medical school and 3 under review medical school respond, making for 18/29 = 0.62 Regarding the paid work, this was freely available information and therefore not restricted by any privacy policy, we also believed it raised interesting questions for which medical schools were to an extent encouraging work during strike action through financial compensation. For previous statements it would have been too much to list every name of medical schools for each statistic, so we had reserved this for the most intriguing one in our own opinion. We do take this on board however and have now anonymised the information which should soon be published as an updated version.

**Reviewer comments:**

***Discussion:*** The discussion and more specifically content in the second/fifth paragraphs was to highlight the need for clear guidance due to potential external pressures to work beyond their capabilities as highlighted by our review of literature. We are not attempting to comment on whether or not students get involved in hospital activities during strike action, but rather that there is clear guidance so that to whatever degree students intend to get involved, they can be protected to an extent from being put in situations that could lead to patient harm and potential damage to their future careers.

**Response: **Regarding paragraph 6, by us pointing out possible conflicts between placement-issued guidance and medical school guidance, we are not alluding to new findings but rather highlighting the 3 placements who offered paid work and stipulating whether this was in complete concordance with the guidance issued from that particular medical school. We are aiming to draw attention that there are several positions of authority commenting on the role medical students could play but it isn't clear who is ultimately responsible for the student.  In terms of safeguarding, in our research there were some discrepancies present between guidance from medical schools and guidance from clinical placements. The finding of potential pressure to act up was based on literature review. 

**Reviewer comments: **The issue of who is ultimately responsible for a paid final-year student if something goes awry - noting that the student is GMC-registered (provisional), 'employed by the trust' and still under the aegis of a medical school is comprehensively discussed in this paragraph. We agree, medical schools are responsible; but in these unprecedented circumstances, it is important to debate the potential responsibility of each organisation. Technically speaking, medical schools hold institutional responsibility until the end of FY1, but the students themselves are ultimately responsible for their own actions (and it would be viewed this way in a fitness-to-practice panel). 

**Response: **An amendment has been made as we agree these findings are preliminary and should hopefully prompt further investigation by medical schools and MedEd researchers alike to provide a better framework for students in such situations.
